# Genetic and epigenetic landscape of IDH-wildtype glioblastomas with *FGFR3*-*TACC3* fusions

**DOI:** 10.1186/s40478-020-01058-6

**Published:** 2020-11-09

**Authors:** Douglas A. Mata, Jamal K. Benhamida, Andrew L. Lin, Chad M. Vanderbilt, Soo-Ryum Yang, Liliana B. Villafania, Donna C. Ferguson, Philip Jonsson, Alexandra M. Miller, Viviane Tabar, Cameron W. Brennan, Nelson S. Moss, Martin Sill, Ryma Benayed, Ingo K. Mellinghoff, Marc K. Rosenblum, Maria E. Arcila, Marc Ladanyi, Tejus A. Bale

**Affiliations:** 1 Department of Pathology, Memorial Sloan Kettering Cancer Center, New York, NY 10065 USA; 2 Department of Neurology, Memorial Sloan Kettering Cancer Center, New York, NY 10065 USA; 3 The Human Oncology and Pathogenesis Program, Memorial Sloan Kettering Cancer Center, New York, NY 10065 USA; 4 Department of Neurosurgery, Memorial Sloan Kettering Cancer Center, New York, NY 10065 USA; 5 Division of Pediatric Neurooncology, German Cancer Consortium, German Cancer Research Center and Hopp Children’s Cancer Center, Heidelberg, Germany

**Keywords:** *FGFR3*-*TACC3* fusion, IDH-wildtype glioblastoma, F3T3

## Abstract

A subset of glioblastomas (GBMs) harbors potentially druggable oncogenic *FGFR3*-*TACC3* (F3T3) fusions. However, their associated molecular and clinical features are poorly understood. Here we analyze the frequency of F3T3-fusion positivity, its associated genetic and methylation profiles, and its impact on survival in 906 IDH-wildtype GBM patients. We establish an F3T3 prevalence of 4.1% and delineate its associations with cancer signaling pathway alterations. F3T3-positive GBMs had lower tumor mutational and copy-number alteration burdens than F3T3-wildtype GBMs. Although F3T3 fusions were predominantly mutually exclusive with other oncogenic RTK pathway alterations, they did rarely co-occur with *EGFR* amplification. They were less likely to harbor *TP53* alterations. By methylation profiling, they were more likely to be assigned the mesenchymal or RTK II subclass. Despite being older at diagnosis and having similar frequencies of *MGMT* promoter hypermethylation, patients with F3T3-positive GBMs lived about 8 months longer than those with F3T3-wildtype tumors. While consistent with IDH-wildtype GBM, F3T3-positive GBMs exhibit distinct biological features, underscoring the importance of pursuing molecular studies prior to clinical trial enrollment and targeted treatment.

**Electronic supplementary material:**

The online version of this article (10.1186/s40478-020-01058-6) contains supplementary material, which is available to authorized users.

## Introduction

The molecular landscape of IDH-wildtype glioblastoma (GBM) has been extensively characterized, yet it remains a disease with a dismal prognosis [8, 9, 21]. Oncogenic fusions have recently been recognized as molecular drivers in a subset of IDH-wildtype GBMs. Approximately 3% of IDH-wildtype GBMs have been reported to harbor activating fusions involving the tyrosine kinase domain (TKD) of the fibroblast growth factor receptor 3 (*FGFR3*) gene and the coiled-coil domain of the transforming acidic coiled-coil-containing protein 3 (*TACC3*) gene [39]. The FGFR3-TACC3 (F3T3) fusion protein is thought to promote malignant transformation by increasing downstream signaling through the MAPK pathway, activating mitochondrial biogenesis and metabolism, and recruiting endogenous TACC3 away from the mitotic spindle, leading to delayed mitotic progression and aneuploidy [20, 37].

The relative rarity of F3T3-positive, IDH-wildtype GBM has hampered a full characterization of this molecular subset of GBM. While prior reports have suggested that these tumors may have recurrent histologic features and molecular profiles, including the absence of *EGFR* amplification and an increased frequency of *CDK4* and *MDM2* amplifications, extensive genomic characterization is lacking, particularly in the context of long-term clinical follow up and survival [7]. Further, since the discovery of F3T3 in GBM, DNA methylation-based tumor classification has emerged as a promising modality for improving diagnostic precision in neuropathology [10]. However, methylation profiles specific to F3T3-positive GBMs have not been reported. Further study of whether F3T3 fusions are associated with other genetic or epigenetic alterations may refine tumor subclassification efforts and impact prognosis and treatment.

The objective of this study was to describe the genomic landscape and methylation profiles of an unbiased institutional cohort of patients with F3T3-positive, IDH-wildtype GBMs and to identify potential associations with patient clinicopathologic characteristics and survival.

## Materials and methods

### Design, setting, and participants

We performed a retrospective cohort study of all patients diagnosed with IDH-wildtype GBMs between January 2015 and December 2019 at Memorial Sloan Kettering Cancer Center (MSK) in New York, NY. The study was approved by the MSK Institutional Review Board and reported in accordance with the STROBE (Strengthening the Reporting of Observational Studies in Epidemiology) guidelines [18].

### Eligibility criteria

All patients with F3T3-positive GBMs were included in the study. All patients with F3T3-wildtype GBMs from the same period were selected for comparison. Cases with *IDH1* or *IDH2* mutations curated as oncogenic by the MSK OncoKB Precision Oncology Knowledge Base were excluded [14].

### Diagnostic criteria

Cases were diagnosed by board-certified MSK neuropathologists (M.K.R., T.A.B.) according to the diagnostic criteria specified in the 2016 World Health Organization Classification of Tumors of the Central Nervous System [28].

### Systematic review and meta-analysis of *FGFR3*-*TACC3* fusion prevalence

The prevalence of F3T3 positivity identified among the IDH-wildtype GBMs by MSK-IMPACT (Integrated Mutation Profiling of Actionable Cancer Targets) in this study was synthesized with previously published estimates using random effects meta-analysis according to a previously reported method [31, 36].

### DNA-based molecular analyses

To assess for the presence of *FGFR3* fusions and other molecular alterations, tumors and matched normal blood samples were analyzed with the MSK-IMPACT next-generation DNA sequencing platform that targets up to 468 genes and select introns to produce data on single nucleotide variants, small insertions and deletions, copy number variation, and structural variants [16, 42]. Tumor mutational burden (TMB) was defined as the number of mutations per megabase (mt/Mb).

### Methylation-based molecular analyses

A subset of cases was analyzed with the Infinium MethylationEPIC (850K) platform, which provides *MGMT* promoter hypermethylation status as well as data on > 850,000 CpG methylation sites across the genome [3, 6]. Cases were assigned methylation-based classes using version 0.1.124 of the random forest-based mnp.v11b4 R package obtained from the German Cancer Research Center (DKFZ) [10]. To investigate possible differences in methylation profiles between F3T3-positive and F3T3-wildtype GBMs, dimensionality reduction with principal component analysis and t-distributed stochastic neighbor embedding was performed and overlaid on a reference cohort of GBMs from the previously published DKFZ study using version 0.15 of the Rtsne package with the following non-default parameters: initial_dims = 100, max_iter = 1500, and theta = 0. Cross-reactive, sex-chromosome, and failed probes were excluded from these analyses.

### Copy number alteration-based cytogenetic analyses

In addition to providing data on methylation profiles, the Infinium platform provides high resolution copy-number variant data similar to that provided by conventional whole-genome copy-number microarray. The chromosome- and arm-level cytogenetic profiles of the GBMs with F3T3 fusions were assessed using version 1.20.0 of the conumee-based MNPcnvplot function in the mnp.v11b4 package in R [26]. The overall tumor copy-number alteration burden (TCB), defined as the percentage of the analyzed genome for which the copy number was not equal to two, was also assessed using the larger MSK-IMPACT dataset. Tumor ploidy was also inferred from the tumor and matched germline MSK-IMPACT sequencing data using the previously validated FACETS algorithm [38].

### Clinical data and survival analyses

Clinical charts were reviewed to extract data on age, sex, date of initial pathologic diagnosis, survival time, history of radiotherapy with or without concurrent temozolomide (TMZ), and treatment history. Reported *MGMT* promoter hypermethylation status, typically determined by pyrosequencing, was extracted from the electronic medical record for those patients who did not undergo methylation analysis on the Infinium platform [32]. Overall survival was defined as the time from diagnostic biopsy until the time of death due to any cause.

### Statistical analyses

Differences in sample means were assessed using the unpaired Student’s t test. Differences in sample medians were assessed using the unpaired Mann–Whitney–Wilcoxon test. Differences among categorical variables were assessed using Fisher’s exact test. Binomial proportion confidence intervals were calculated using the Clopper-Pearson method. Clinical and pathological variables were examined in univariate and multivariate Cox proportional hazards models for associations with overall survival. The multivariate models were adjusted for potential confounders including age, sex, race, and *MGMT* status. Genomic data were accessed using the internal MSK cBioPortal for Cancer Genomics and statistical analyses were performed using R version 3.6.2 (R Foundation for Statistical Computing) [13, 22, 35]. Statistical tests were 2-sided and used a significance threshold of *p* < 0.05. Reported *p* values were not adjusted for multiple testing.

## Results

### Description of the cohort

**Fig. 1:**
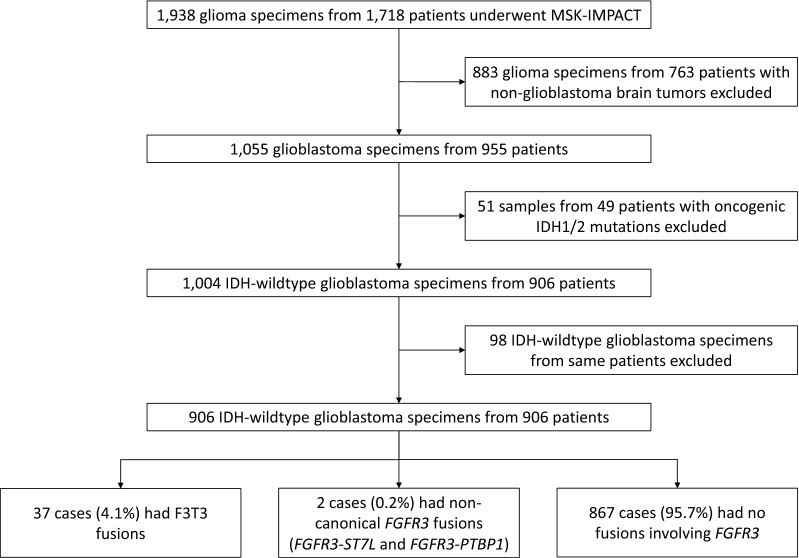
Flow diagram of specimen selection. *Note*: The two tumors with non-canonical fusions were excluded from further analyses because dimerization domains have not been reported in these 3′ partner genes [44]

Between January 2015 and December 2019, samples from 906 patients with IDH-wildtype GBMs underwent DNA sequencing with MSK-IMPACT (Fig. 1). In all, 4.1% (37/906) exhibited F3T3 fusions, 0.2% (2/906) exhibited non-canonical fusions involving *FGFR3* (one each of *FGFR3*-*ST7L* and *FGFR3*-*PTBP1*, which are excluded from further analyses since dimerization domains have not been reported in these 3′ partner genes [44]), and 95.7% (867/906) had no fusions involving *FGFR3*. The F3T3 gene fusions most commonly involved *FGFR3* exons 17 (67.6% [25/37]) or 18 (29.7% [11/37]) and *TACC3* exons 11 (37.8% [14/37]) or 10 (24.3% [9/37]) (Additional file 1: Fig. S1).

Patients with F3T3-positive tumors in this cohort were significantly older at diagnosis, with a mean age of 63.6 years (interquartile range [IQR], 57.2–72.2) compared to 59.4 years (IQR, 52.0–68.1) for patients with F3T3-wildtype tumors (*p* = 0.02) (Table 1). The sex composition of the two groups was similar (40.5% [15/37] vs. 39.6% [343/867] female, *p* = 0.96). There was no difference in laterality or anatomic localization (*p* > 0.05 for both comparisons). All tumors were reviewed by a board-certified neuropathologist and found to meet histologic criteria for GBM, WHO grade IV (i.e., highly cellular and variably anaplastic glial cells with brisk mitotic activity, microvascular proliferation, and/or necrosis) [28].

**Table 1:** Clinical and imaging characteristics of patients with glioblastomas with and without *FGFR3*-*TACC3* fusions

	*FGFR3*-*TACC3* fusion		*P*
	Present (n = 37)	Absent (n = 867)	
*Age*			0.02
Mean (IQR)	63.6 (57.2–72.2)	59.4 (52.0–68.1)	
*Sex*			1.00
Male	22 (59.5%)	524 (60.4%)	
Female	15 (40.5%)	343 (39.6%)	
*Ethnicity*			0.32
White	33 (89.2%)	718 (89.0%)	
Black	3 (8.1%)	26 (3.2%)	
Asian	1 (2.7%)	56 (6.9%)	
Hispanic	0 (0%)	7 (0.9%)	
Unavailable	0	60	
*Laterality*			0.72
Left	14 (45.2%)	313 (49.1%)	
Right	17 (54.8%)	325 (50.9%)	
Unavailable	6	229	
*Location*			0.68
Frontal	13 (35.1%)	222 (33.9%)	
Temporal	11 (29.7%)	196 (30.0%)	
Parietal	9 (24.3%)	153 (23.4%)	
Occipital	2 (5.4%)	41 (6.3%)	
Callosal	2 (5.4%)	12 (1.8%)	
Cerebellar	0 (0%)	4 (0.6%)	
Other	0 (0%)	26 (4.0%)	
Unavailable	0	213	

### Prevalence of *FGFR3*-*TACC3* fusions compared to previously published studies of IDH-wildtype GBMs

**Fig. 2:**
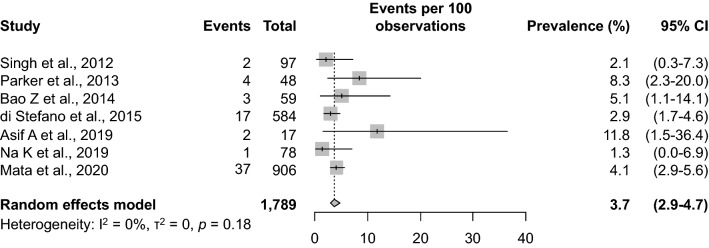
Systematic review and meta-analysis of *FGFR3*-*TACC3* fusion prevalence among IDH-wildtype glioblastomas. *Note*: The vertical dashed line indicates the pooled summary estimate (95% confidence interval [CI]) for all studies. The area of each square is proportional to the inverse variance of the estimate. Horizontal lines indicate 95% CIs of the estimate. The studies are listed in chronological order of publication

The 4.1% (37/906) prevalence of F3T3 fusions identified in this study was compared to prevalence estimates reported in six previously published studies [2, 5, 17, 33, 34, 39]. Taken together, the previously published studies involved a total of 883 IDH-wildtype GBMs (median, 68.5; range 17–584). Meta-analytic pooling of the prevalence value from the present study with those from the six previously published studies yielded a summary F3T3-fusion prevalence of 3.7% (95% confidence interval [CI], 2.9–4.7%), with no evidence of between-study heterogeneity (Q = 8.8, τ^2^ = 0, *I*^2^ = 0%, *p* = 0.18) (Fig. 2). The prevalence estimates reported by the individual studies ranged from 1.3 to 11.8%. Sensitivity analysis, in which the meta-analysis was serially repeated after exclusion of each study, demonstrated that no individual study affected the overall estimate by more than 0.4%. Thus, we conclude that the prevalence of F3T3 fusions in IDH-wildtype GBMs is approximately 4%.

### Associations between *FGFR3*-*TACC3* fusions and canonical drivers of glioblastoma tumorigenesis

**Fig. 3:**
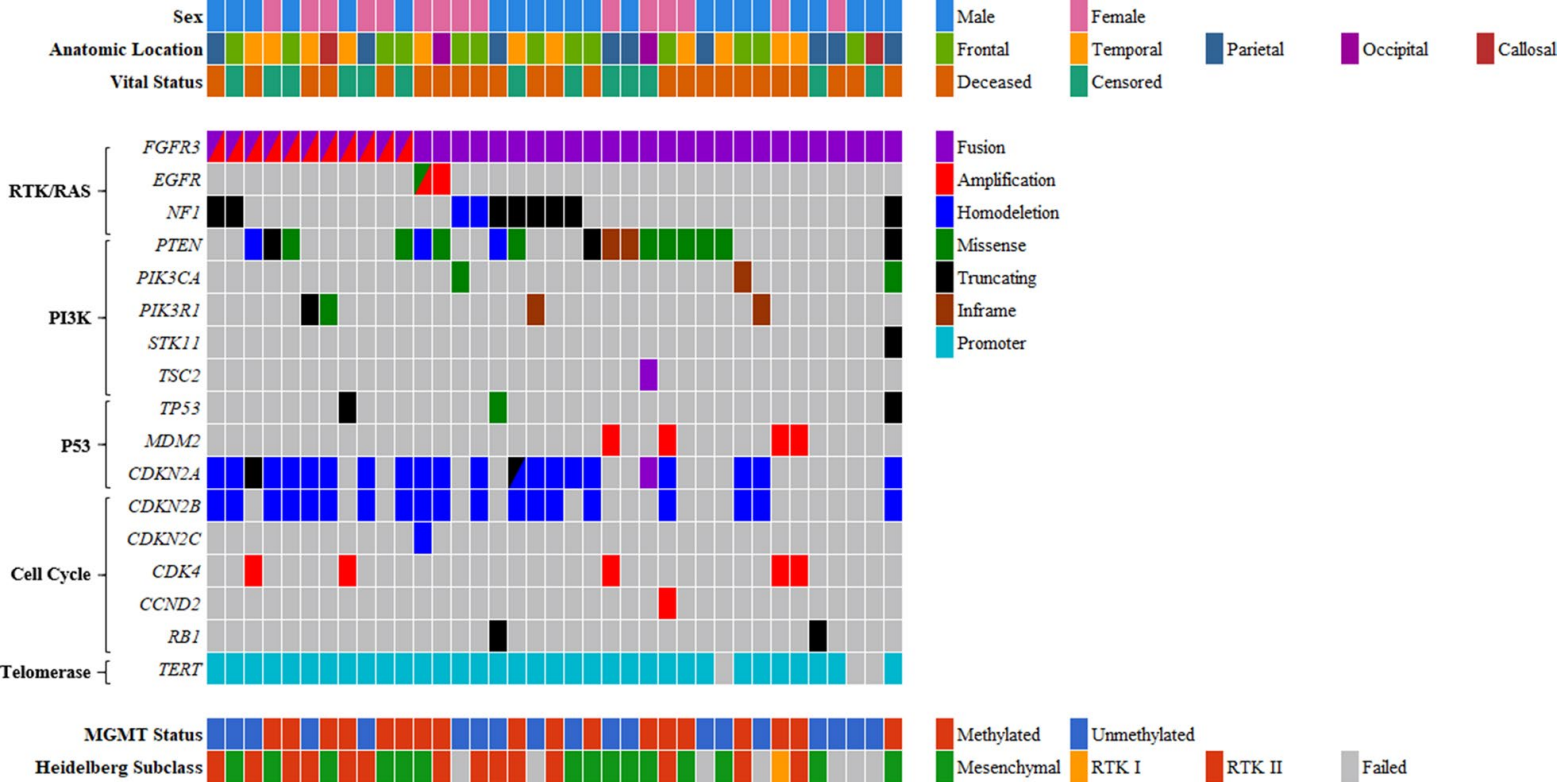
Clinical characteristics, cancer signaling pathway alterations, and methylation profiles of 37 *FGFR3*-*TACC3* fusion-positive glioblastomas. *Note*: The variants displayed are putative drivers according to OncoKB and/or the Cancer Hotspots database [14, 15]

**Fig. 4:**
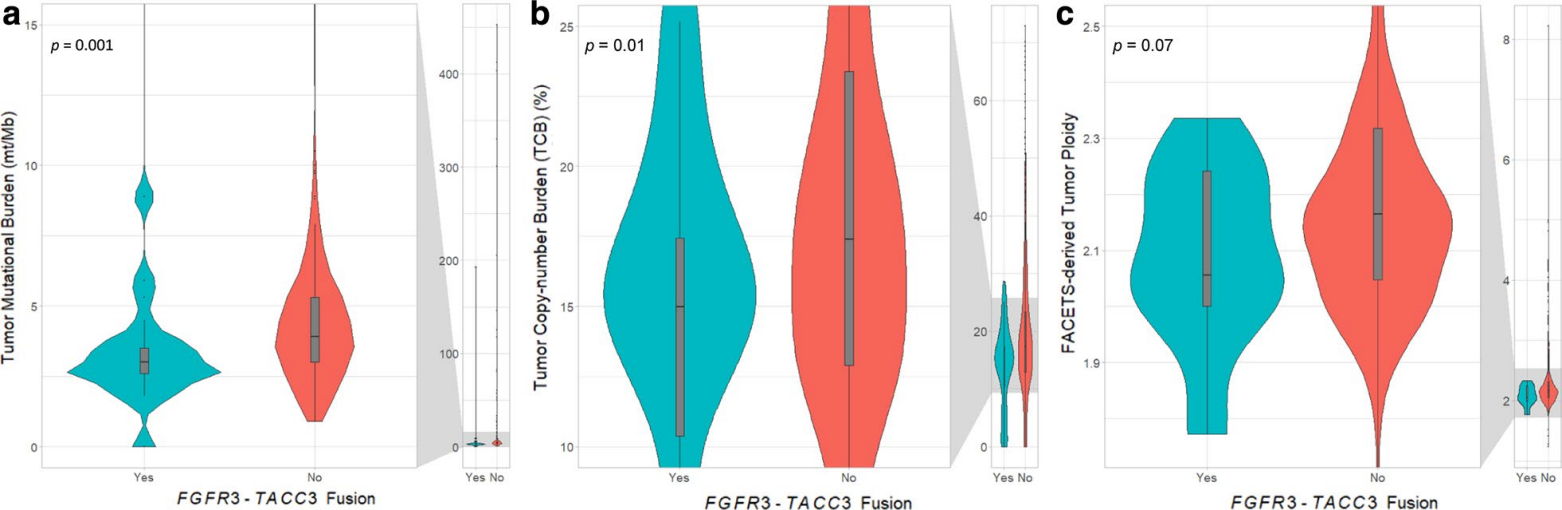
Tumor mutational burden (**a**), copy-number burden (**b**), and FACETS-derived tumor ploidy (**c**) stratified by *FGFR3*-*TACC3* fusion status. *Note*: The violin plot is a box plot with a rotated kernel density plot on each side. The plot allows the reader to visualize the median, interquartile range, range, and distribution of the data

The relative frequencies of selected mutations and copy-number alterations curated as potentially oncogenic in OncoKB [14] and/or as putative driver alterations in the Cancer Hotspots [15] database in the RTK/RAS/MAPK, PI3K, P53, cell cycle, and telomere maintenance pathways among the 37 F3T3-positive GBMs are presented in Fig. 3 and Additional file 1: Fig. S2. F3T3-positive tumors had significantly lower median TMB than F3T3-wildtype GBMs (3.0 mt/Mb [IQR, 2.6–3.5] vs. 3.9 mt/Mb [IQR, 3.0–5.3], *p* = 0.001), a difference that persisted after exclusion of cases with hypermutation (TMB ≥ 20 mt/Mb) secondary to TMZ therapy (Fig. 4a).

**Fig. 5:**
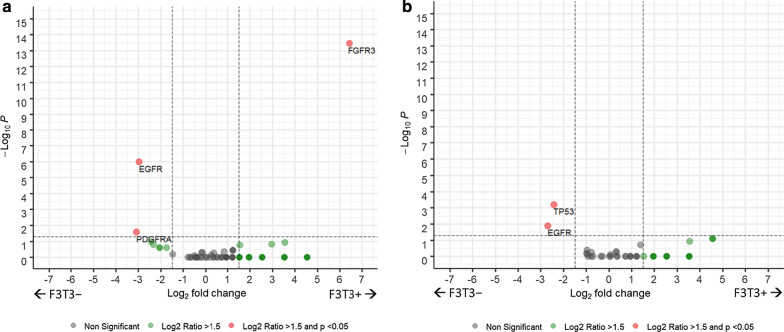
Differences in copy-number alterations (**a**) and mutations (**b**) among glioblastomas with and without *FGFR3*-*TACC3* fusions. Legend: The scatterplot shows statistical significance (−log_10_ of *p* value) versus magnitude of change (log_2_ of fold change) for copy-number altered (**a**) and mutated (**b**) genes. The horizontal dashed line indicates a *p* value of 0.05. The vertical dashed lines indicate fold changes of 1.5. Positive fold changes indicate that the alterations are more common in *FGFR3*-*TACC3* positive tumors, and vice versa

Between-group differences in oncogenic somatic mutations and copy-number alterations among the non-hypermutated F3T3-positive (n = 36) and F3T3-wildtype (n = 844) cases were compared (Fig. 5). F3T3 fusions were predominantly mutually exclusive with other oncogenic alterations in the RTK pathway. F3T3-positive cases were less likely to exhibit concurrent *EGFR* amplification (5.6% [2/36] vs. 43.4% [366/844], *p* < 0.001) or mutation (2.8% [1/36] vs. 18.1% [153/844], *p* < 0.01) and were mutually exclusive with *PDGFRA* (0% [0/36] vs. 11.6% [98/844], *p* = 0.03), *KIT* (0% [0/36] vs. 7.5% [63/844], *p* = 0.10), and *MET* amplification (0% [0/36] vs. 2.1% [18/844], *p* = 1.0). Conversely, they were more likely to exhibit concurrent *FGFR3* amplification (30.6% [11/36] vs. 0.4% [3/844], *p* < 0.001). An additional 52.8% (19/36) had evidence of low-level gains in *FGFR3* consistent with the tandem duplication event that forms the F3T3 fusion product [34].

The proportion of downstream RAS/MAPK alterations in the F3T3-positive and F3T3-wildtype groups was also examined. There was no significant difference in the frequency of *NF1* inactivation (25.0% [9/36] vs. 17.2% [145/844], *p* = 0.26). *BRAF* mutations (0% [0/36] vs. 2.6% [22/844], *p* = 1.0) were not observed in F3T3-positive tumors. Similarly, oncogenic alterations in *KRAS*, *NRAS*, *HRAS*, *RIT1*, *RASA1*, and *RAF1* were not identified in any of the F3T3-positive tumors and were only very rarely identified (< 1.0% of cases) in F3T3-wildtype tumors (*p* = 1.0 for all comparisons). The relative proportion of PI3K pathway alterations was similar, including the frequency of *PIK3CA* mutations (5.6% [2/36] vs. 9.5% [80/844], *p* = 0.57), *PIK3R1* mutations (11.1% [4/36] vs. 8.9% [75/844], *p* = 0.56), and *PTEN* inactivation (44.4% [16/36] vs. 46.7% [394/844], *p* = 0.87).

F3T3-positive tumors were less likely to harbor certain P53 pathway alterations than F3T3-wildtype tumors. Specifically, F3T3-positive cases were significantly less likely to exhibit concurrent oncogenic alterations in *TP53* (5.6% [2/36] vs. 30.9% [261/844], *p* < 0.001) and were mutually exclusive with amplifications in *MDM4* (0% [0/36] vs. 6.9% [58/844], *p* = 0.16). The frequencies of *MDM2* amplification (11.1% [4/36] vs. 8.5% [72/844], *p* = 0.54) and *CDKN2A* inactivation (55.6% [20/36] vs. 60.3% [509/844], *p* = 0.60) were similar.

The frequencies of cell cycle pathway alterations between the F3T3-positive and F3T3-wildtype groups did not substantially differ, including for *RB1* inactivation (5.6% [2/36] vs. 13.2% [111/844], *p* = 0.30), *CDKN2B* inactivation (50.0% [18/36] vs. 56.9% [480/844], *p* = 0.49), *CDKN2C* inactivation (2.8% [1/36] vs. 4.1% [35/844], *p* = 1.0), *CCND2* amplification (2.8% [1/36] vs. 1.5% [13/844], *p* = 0.44), *CDK4* amplification (13.9% [5/36] vs. 13.9% [117/844], *p* = 1.0), and *CDK6* amplification (0% [0/36] vs. 1.9% [16/844], *p* = 1.0).

There was no significant difference in the frequency of *TERT* promoter mutations (91.7% [33/36] vs. 88.2% [744/844], *p* = 0.79) between the F3T3-positive and F3T3-wildtype groups. Last, no F3T3-positive cases exhibited *ATRX* (0% [0/36] vs. 2.7% [23/844], *p* = 0.62) or *H3F3A* mutations (0% [0/36] vs. 1.7% [14/844], *p* = 1.0).

### Methylation profiles of glioblastomas with and without *FGFR3*-*TACC3* fusions

**Fig. 6:**
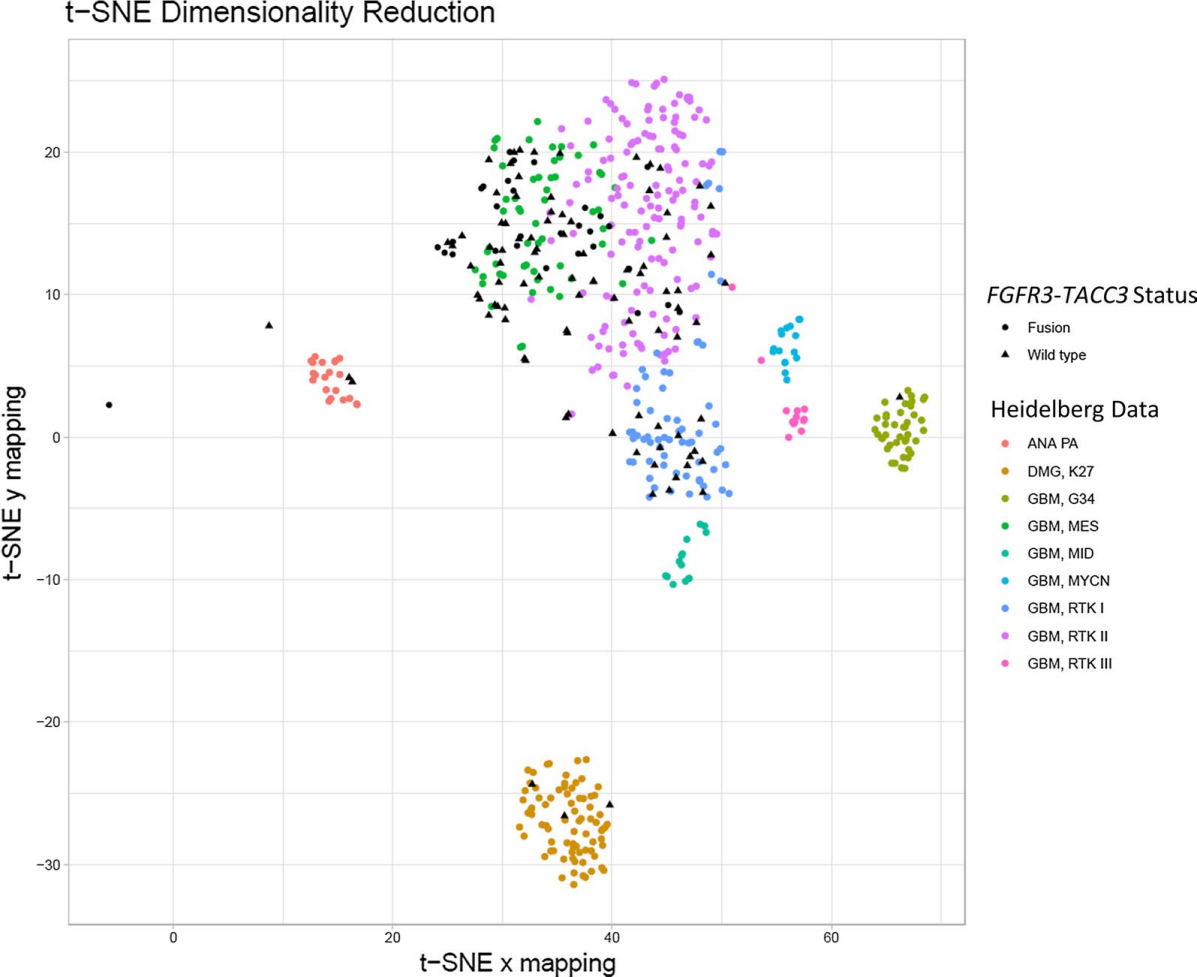
Dimensionality reduction demonstrated that *FGFR3*-*TACC3* fusion-positive tumors were more likely to be assigned to the mesenchymal and RTK II methylation subclasses. *Note*: Cases were assigned DNA methylation-based classifications according to the method described by Capper et al. [10]. F3T3-positive GBMs overwhelmingly fell into the GBM, MES and GBM, RTK II clusters

A subset of the F3T3-positive (91.9% [34/37]) and -wildtype (11.5% [100/867]) GBMs was analyzed on the Infinium platform [3] and classified using the Heidelberg methylation-based glioma classification tool [10]. There was no difference in the frequency of *MGMT* promoter hypermethylation between the F3T3-positive and F3T3-wildtype cases (52.9% [18/34] vs. 48.0% [48/100], *p* = 0.69). The Heidelberg tool confidently assigned classes to 88.2% (30/34) of the F3T3-positive tumors and 93.0% (93/100) of the F3T3-wildtype tumors (*p* = 0.47). F3T3-positive tumors were more likely to be assigned the mesenchymal or RTK II subclass (grouped together for this analysis due to their overlapping methylation characteristics) than were F3T3-wildtype tumors (96.7% [29/30] vs. 72.0% [67/93], *p* = 0.004) (Additional file 1: Table S1). The F3T3-positive mesenchymal and RTK II subclass tumors did not significantly differ with respect to median TMB (3.0 mt/Mb [IQR, 2.6–3.5] vs. 3.5 mt/Mb [IQR, 2.6–4.5], *p* = 0.93) or in *TP53* alteration frequency (6.7% [1/15] vs. 14.3% [2/14], *p* = 0.60). Only one F3T3-positive case was assigned the RTK I subclass (3.3% [1/30]). In contrast, 14.3% (5/35) of *EGFR*-amplified cases tested and 50.0% (6/12) of *PDGFRA*-amplified cases tested were assigned the RTK I subclass. When stratified by F3T3-fusion status and considered alongside 446 previously published GBM methylation profiles from the Heidelberg cohort, dimensionality reduction with principal component analysis and t-distributed stochastic neighbor confirmed these findings (Fig. 6). F3T3-positive tumors were 2.0 × more likely (95% CI, 1.8–2.2, *p* < 0.0001) to be assigned to the mesenchymal or RTK II subclass and 0.1 × (95% CI, 0.01–0.5, *p* = 0.01) as likely to be assigned to any other subclass.

### Copy number alteration-based cytogenetic analysis of glioblastomas with and without *FGFR3*-*TACC3* fusions

Among F3T3-positive tumors with analyzable data, 78.1% (25/32) had concurrent chromosome 7 gain and 10q loss, 9.4% (3/32) had chromosome 7 gain without 10q loss, 6.3% (2/32) had 10q loss without chromosome 7 gain, and 6.3% (2/32) had concurrent loss of 10q, 13q, and 14q, all consistent with the integrated diagnosis of IDH-wildtype GBM. The overall TCB was assessed using the larger MSK-IMPACT dataset that included all 904 patients. Because the F3T3 fusion protein is thought to cause aneuploidy, we hypothesized that F3T3-positive GBMs would have higher TCBs than F3T3-wildtype GBMs. Unexpectedly, F3T3-positive tumors had significantly lower TCBs. The median TCB for the F3T3-positive tumors was 15.0% (IQR, 10.4–17.5%) while that for the F3T3-wildtype tumors was 17.4% (IQR, 12.9–23.4%) (*p* = 0.006) (Fig. 4b). A similar finding was obtained using a subset (n = 371) of the MSK-IMPACT paired germline data in an allele-specific copy-number analysis of tumor ploidy using the FACETS algorithm [38], which revealed that F3T3-positive tumors had slightly lower median ploidy (2.06 × [IQR, 2.00–2.24] vs. 2.16 × [IQR, 2.05–2.32], *p* = 0.07) (Fig. 4c).

### Clinical characteristics and overall survival

**Fig. 7:**
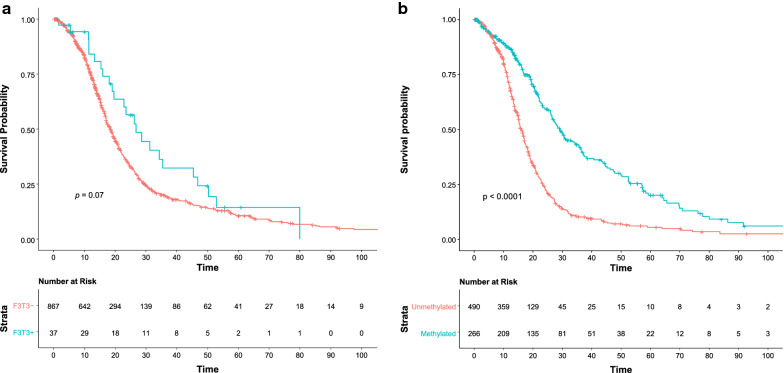
Kaplan-Meier analysis of overall survival stratified by *FGFR3*-*TACC3* fusion (**a**) and *MGMT* promoter hypermethylation (**b**) status. *Note*: Overall survival was defined as the time from initial diagnostic biopsy until the time of death due to any cause. Two-sided *p* values for difference were calculated using the log-rank test

Follow-up data were sought for all patients through a comprehensive medical-record and obituary search. The median follow-up time was 55.6 months (IQR, 24.9–60.9) among F3T3-positive and 52.0 months (IQR, 19.8–92.9) among F3T3-wildtype patients (*p* = 0.9). In all, 64.9% (24/37) of F3T3-positive and 71.5% (620/867) of F3T3-wildtype patients died during follow up (*p* = 0.36). On univariate Kaplan–Meier analysis, F3T3-positive patients lived slightly longer than F3T3-wildtype patients (26.7 [IQR, 16.0–46.9] vs. 18.5 [IQR, 12.1–29.7] months, *p* = 0.07), corresponding to a hazard ratio of 0.69 (95% CI, 0.46–1.04) (Fig. 7). As expected, *MGMT* promoter hypermethylation was associated with improved survival on univariate analysis (29.2 [IQR, 17.2–57.1] vs. 16.3 [IQR, 11.3–23.5] months, *p* < 0.0001), corresponding to a hazard ratio of 0.43 (95% CI, 0.36–0.53). In a multivariable model adjusted for age, sex, race, and *MGMT* status, the hazard ratio for F3T3 positivity was 0.68 (95% CI 0.45 to 1.03, *p* = 0.07), similar to the association identified on univariate analysis.

### Treatment of patients with *FGFR3*-*TACC3* fusions

Treatment data were available for 91.9% (34/37) of the patients with F3T3-positive GBMs, all of whom received upfront radiotherapy and TMZ (concurrently and/or as adjuvant therapy). Only a minority of patients received a therapy targeting FGF/FGFR signaling. Specifically, 5.9% (2/34) received a selective FGFR inhibitor and 5.9% (2/34) received a multikinase inhibitor/non-selective FGFR inhibitor. The small number of patients receiving targeted therapy precluded investigating its association with overall survival.

## Discussion

This genomic landscape study of 906 patients with IDH-wildtype GBMs treated at a tertiary cancer referral center demonstrated that 4.1% harbored potentially druggable activating F3T3 fusions with recurrent structural isoforms; characteristic associated mutational, copy-number, and methylation profiles; and clinical outcomes slightly better than patients with F3T3-wildtype tumors despite occurring in older individuals.

Our understanding of F3T3-positive GBMs has increased since the initial discovery that demonstrated F3T3 fusions comprised a rare yet recurrent oncogenic structural event resulting in an in-frame fusion protein with constitutive kinase activity and mitogenic effects, which were reversible by FGFR kinase inhibition [34, 39]. Together with subsequent studies involving a total 883 IDH-wildtype GBMs, it was previously estimated that 1–12% might harbor F3T3 fusions, with 3% a commonly cited prevalence estimate [2, 5, 17, 33, 34, 39]. The present study, involving a large, unbiased single-institutional cohort of 906 prospectively sequenced GBMs, revealed an F3T3-fusion prevalence of 4.1% among individuals with IDH-wildtype GBMs. Meta-analytic pooling suggested a similar prevalence, with an upper confidence interval indicating that as many as 5% of GBMs may have F3T3 fusions, slightly higher than suggested by prior consensus. Given ongoing interest in targeted FGFR3 inhibition, these data support the importance of pursuing comprehensive molecular characterization with robust fusion detection on all IDH-wildtype GBMs to identify patients for potential clinical trial enrollment and targeted treatment.

The present study confirms molecular features of F3T3-positive GBMs suggested in prior reports, including the diversity of structural isoforms of the F3T3 fusion gene itself. Prior studies established that isoforms involving *FGFR3* exons 17 or 18 were most common [17, 34, 39], in agreement with our finding that 97.3% (36/37) of cases included one of these exons. As in prior studies, we found great variability for the 3′ *TACC3* exon, ranging from exons 3–12. Overall, *FGFR3* exon 17 to *TACC3* exon 11 and *FGFR3* exon 17 to *TACC3* exon 10 fusions were most common.

However, our data reveal new insights into the molecular alterations associated with F3T3 fusions in IDH-wildtype GBMs, in some instances contrasting with prior smaller studies that employed more targeted sequencing methods. For example, prior reports suggested that the F3T3 fusion was mutually exclusive with *EGFR*, *PDGFRA*, or *MET* amplification [23, 34], and had an increased frequency of co-occurring *CDK4* and *MDM2* amplification [7, 17]. In the present study, F3T3-positivity was indeed mutually exclusive with amplification in *PDGFRA* or *MET*. Similarly, we found that F3T3-positive GBMs were less likely to exhibit concurrent *EGFR* amplification (5.6% vs. 43.3%); however, our data revealed that these events were not truly mutually exclusive, as two cases with F3T3 fusions exhibited *bona fide* amplification in *EGFR*. However, it is possible that the F3T3 fusion and the *EGFR* amplification events were present in different subclones, as has been shown for amplification of diverse RTKs [40, 41]. Further, there was no increased frequency of *CDK4* or *MDM2* amplification among F3T3-positive GBMs in our study.

The present study utilized a hybridization capture-based NGS assay (MSK-IMPACT) for sequencing all exons and selected introns of up to 468 genes with paired tumor-matched normal tissue analysis enabling unambiguous somatic mutation detection [16, 42], allowing us to comprehensively analyze oncogenic pathway alterations. A key negative finding gleaned from these data was a lack of significant differences in downstream PI3K, cell cycle, and telomere maintenance pathway alterations in F3T3-positive and F3T3-wildtype GBMs. Also interesting was the observation that oncogenic alterations in *TP53* and *MDM4* were *less* common among F3T3-positive tumors. Combined with the lower median TMB and TCB observed in F3T3-positive GBMs, these data suggest that F3T3-positivity itself may be enough to drive oncogenesis in the relative absence of other concurrent driver alterations. The relatively genomically quiet nature of the F3T3-positive GBMs was unexpected given that experimental data suggest that the F3T3 fusion protein recruits endogenous TACC3 away from the mitotic spindle, leading to delayed mitotic progression and aneuploidy [37]; our data therefore suggest that additional mechanisms to counteract this activity (e.g., the relative rarity of concurrent *TP53* and *MDM4* alterations identified in this study) may be engaged in vivo.

DNA methylation-based CNS tumor classification is becoming increasingly employed as a diagnostic tool in many neuropathology practices worldwide. The present study provides insight into the F3T3-positive GBM methylome using a previously validated machine-learning-based tumor classifier developed at University Hospital Heidelberg [10]. Aside from confirming the prior observation that *MGMT* promoter hypermethylation does not differ between F3T3-positive and F3T3-wildtype GBMs [17], the present analysis demonstrated that the majority of F3T3-positive GBMs can be accurately classified as IDH-wildtype GBMs by the Heidelberg classifier. Further, it showed that F3T3-positive cases overwhelmingly (in 96.7% of cases) fall into the mesenchymal or RTK II subclasses, which overlap in their methylation profiles, rather than the RTK I (or any other) subclass. In contrast, GBMs with *EGFR* or *PDGFRA* amplifications were assigned the RTK I subclass in 14.3% and 50.0% of cases, respectively, consistent with prior reports [10]. While it is unclear if the role of *TACC3* in regulating the epithelial-mesenchymal transition may relate to the methylation characteristics of F3T3-positive GBM [24], it has been suggested that methylation subclass is reflective of tumor cell of origin as well as somatic epigenetic changes [11, 19, 25]. Our data show that while F3T3-positive GBMs are closely related to other IDH-wildtype GBMs, there are differences among these tumors that may be biologically significant.

The present study is among the first to provide long-term clinical follow-up data on a large cohort of patients with F3T3-positive tumors, with a median follow-up time of 55.6 months. Patients with F3T3-positive tumors were slightly older at initial diagnosis (63.6 vs. 59.4 years). Although older age at diagnosis is a well-established risk factor for poorer outcome and shorter overall survival in patients with GBM, patients with F3T3-positive GBMs in this study lived slightly longer than those without F3T3 fusions (26.7 vs. 18.5 months, *p* = 0.07), a difference that persisted after adjustment for potential confounders including age, sex, race, and *MGMT* status. Although this difference did not meet formal statistical significance, at the very least, we can conclude that patients with F3T3-positive tumors have clinical features and outcomes similar to, or perhaps slightly better than, those with non-F3T3-driven, IDH-wildtype GBMs.

Of note, F3T3 fusions have also been identified in lower-grade histologic entities [17, 23, 27, 29, 43]. Sufficient long-term follow up is lacking to determine if any among these are under-sampled higher-grade gliomas, or gliomas that eventually recur, progress, or otherwise behave aggressively. Furthermore, both low- and high-grade F3T3-positive gliomas exhibit characteristic histologic features, including monomorphous oligodendroglioma-like nuclei, “chicken-wire” capillary networks, and frequent microcalcifications [7]. While these features suggest the attractive hypothesis that F3T3-positive GBMs arise from lower-grade precursor lesions, to date there has been insufficient evidence to support this idea. Rather, our study underscores that F3T3 fusions, when detected in histologic GBM, drive clinical behavior akin to other IDH-wildtype GBMs.

Importantly, given the difference in overall survival suggested by this study, our findings raise the possibility that the underlying biology of F3T3-positive GBM may be a confounding factor in interpreting outcome data in the setting of pan-GBM clinical trials. Considering ongoing research into therapeutic inhibition of tyrosine kinase signaling in general and FGFR3 signaling specifically, identification of F3T3 fusions in patients with IDH-wildtype GBMs could soon have therapeutic implications. For example, the recent phase 2 REGOMA trial showed that median overall survival was approximately 2 months longer in patients with first recurrence of GBM after surgery and radiotherapy/TMZ receiving the oral multikinase inhibitor regorafenib rather than lomustine (7.4 vs. 5.6 months, *p* < 0.001) [30]. Notably, that study did not enroll patients based on a molecular alteration, nor did it report results stratified by underlying somatic genetic alterations, so its relevance to patients with F3T3-positive GBMs is uncertain.

FGFR signaling has been implicated in a variety of human cancers. FGFR pathway inhibition as a therapeutic strategy remains an area of active investigation, including in ongoing and recently completed clinical trials of FGFR inhibitors in brain tumors (e.g., NCT01975701, NCT028224133, NCT02052778, and NCT01948297). Other inhibitors of FGFR3 and F3T3 have also been tested in preclinical models and in trials in other tumor types, especially urothelial carcinoma and cholangiocarcinoma, for which therapeutics such as erdafitinib, rogaratinib, infigratinib, and the monoclonal antibody vofatamab have shown promise [12]. Recent research has also demonstrated that F3T3-positive tumors are characterized by mitochondrial activation, and that these tumors may be particularly susceptible to inhibitors of mitochondrial respiration and oxidative metabolism, highlighting this pathway as another potential therapeutic opportunity [20]. Aside from FGFR3, there is also interest in inhibiting TACC3, including with the potent new TACC3-targeting agent BO-264, which significantly inhibited the growth of cells harboring F3T3 fusions in a preclinical model [1].

This study has limitations. Although 906 cases were sequenced, only 137 underwent methylation array analysis. Also, an insufficient number of F3T3-positive patients in this cohort received F3T3-specific targeted therapy to evaluate the potential efficacy of such an approach, the determination of which awaits future clinical trials. Last, detailed treatment information was not available for all F3T3-wildtype patients, thus chemoradiotherapy treatment status was not adjusted for in the Cox proportional hazards regression models.

In conclusion, approximately 4% of IDH-wildtype GBMs in this study harbored potentially targetable F3T3 fusions. While their clinical and molecular characteristics were largely in keeping with other IDH-wildtype GBMs, they demonstrated characteristic associated mutational, copy-number, and methylation profiles, and patients with F3T3-positive tumors had clinical outcomes slightly better than patients with F3T3-wildtype tumors. F3T3-positivity was predominantly mutually exclusive with other RTK drivers, and F3T3-wildtype tumors were enriched for mutations in *TP53*. As histologic features lack specificity for identifying F3T3-positive tumors, comprehensive NGS and methylation analysis should be considered for all IDH-wildtype GBMs to identify patients for potential clinical trial enrollment and targeted treatment.

## Supplementary information

**Additional file 1**. Online supplementary material.

## Data Availability

The datasets analyzed in the current study are available from the corresponding authors on reasonable request.
