# Supplementary material for: Genetic and epigenetic landscape of IDH-wildtype glioblastomas with FGFR3-TACC3 fusions
Source: Acta Neuropathol Commun. 2020 Nov 9;8:186. doi: 10.1186/s40478-020-01058-6 (PMC7653727; doi:10.1186/s40478-020-01058-6)
Supplement: Supplementary file 1 — Additional file 1. Online supplementary material. [file 40478_2020_1058_MOESM1_ESM.docx]

# Supplementary Figure 1. Structure of *FGFR3*-*TACC3* gene fusions identified by MSK-IMPACT.

**
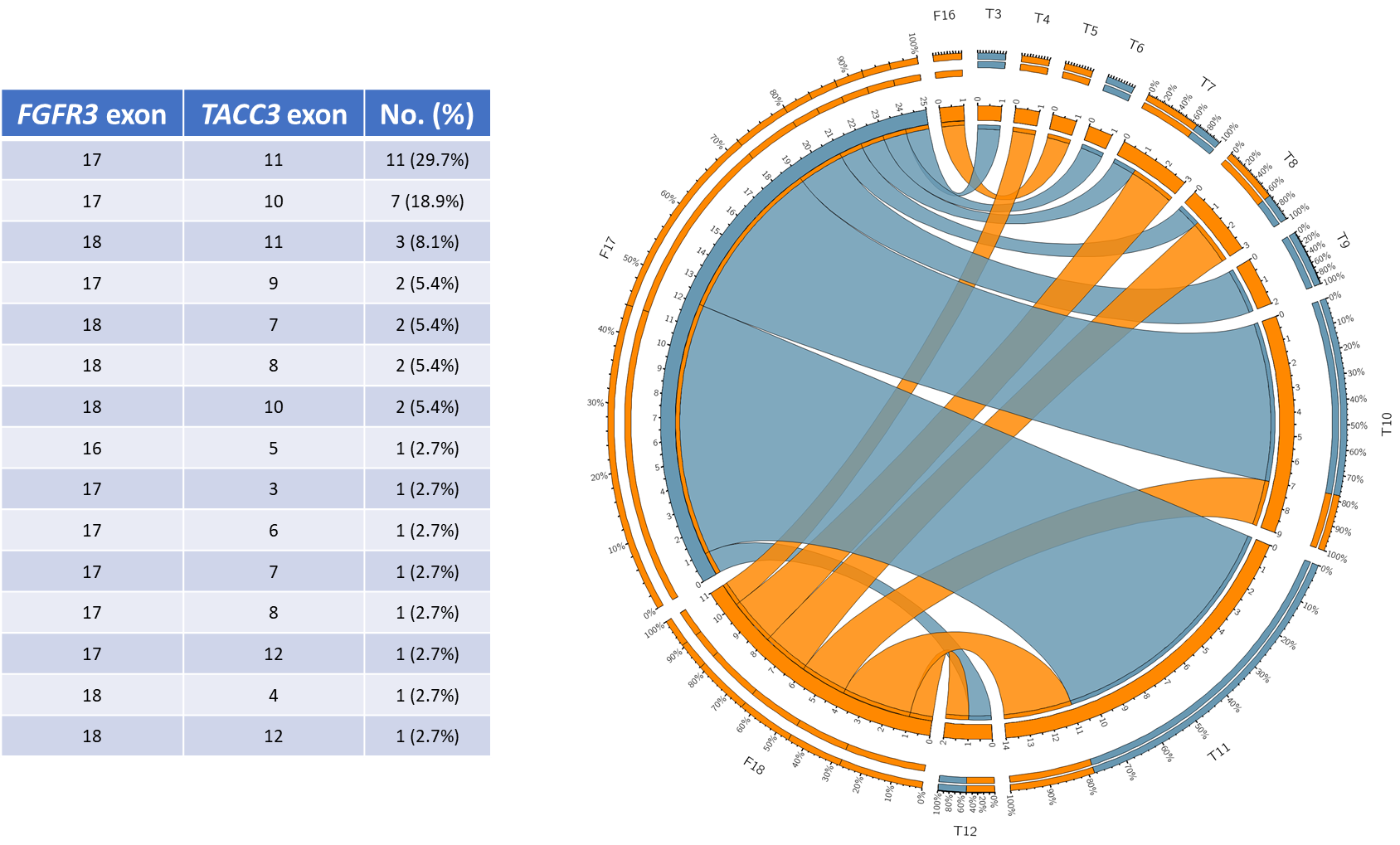
**

**Legend:** The number following the first letter of the gene denotes the exon involved (e.g., F17 denotes *FGFR3* exon 17). The width of the ribbons connecting the 5′ and 3′ gene partners corresponds to the frequency of that exon-to-exon connection.

# Supplementary Figure 2. RTK/RAS/MAPK, PI3K, P53, and cell cycle pathway analysis among 37 *FGFR3*-*TACC3* fusion-positive glioblastomas.

**(A)** RTK/RAS/MAPK and PI3K pathways.


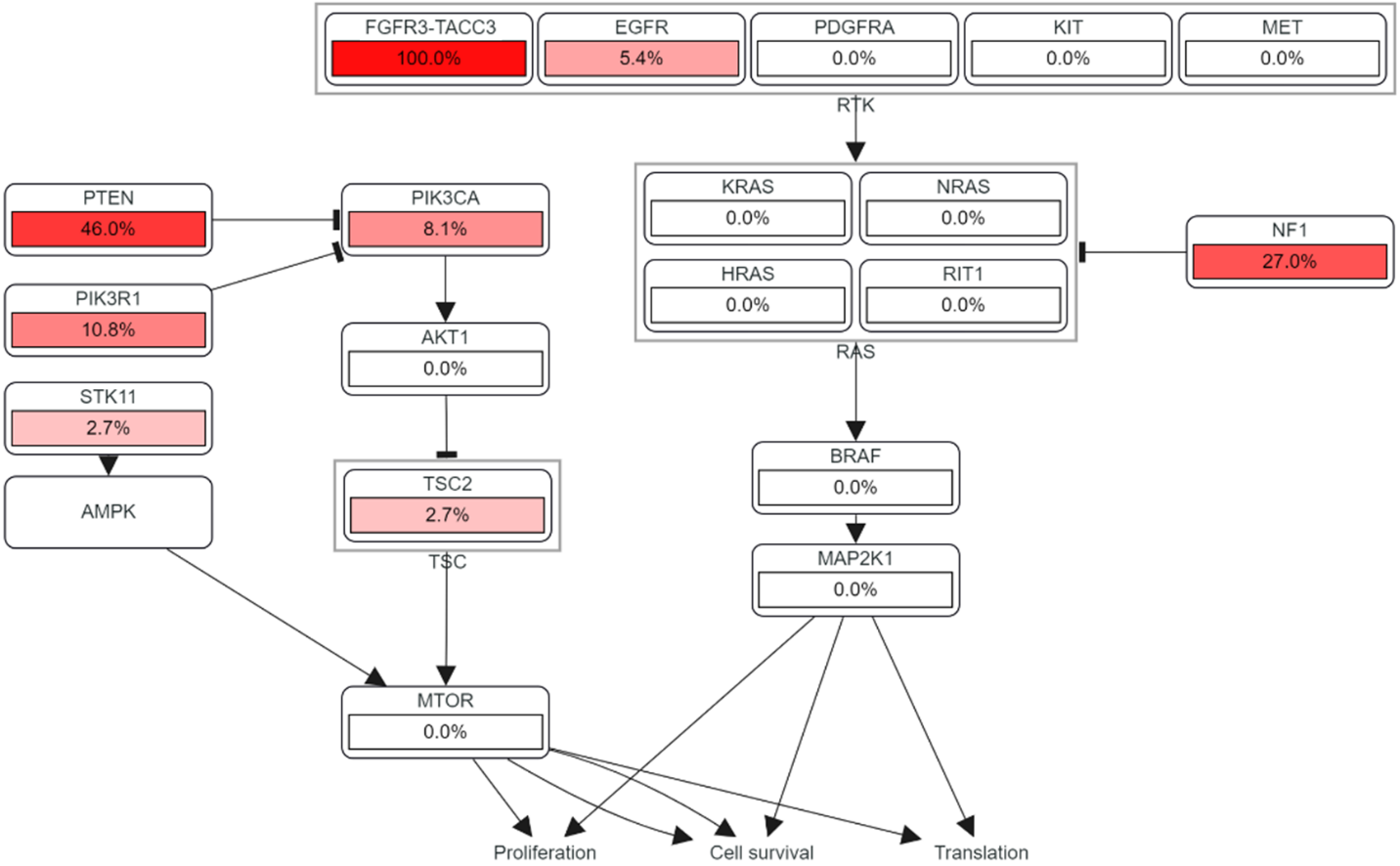


**(B)** P53 pathway. **(C)** Cell cycle pathway.


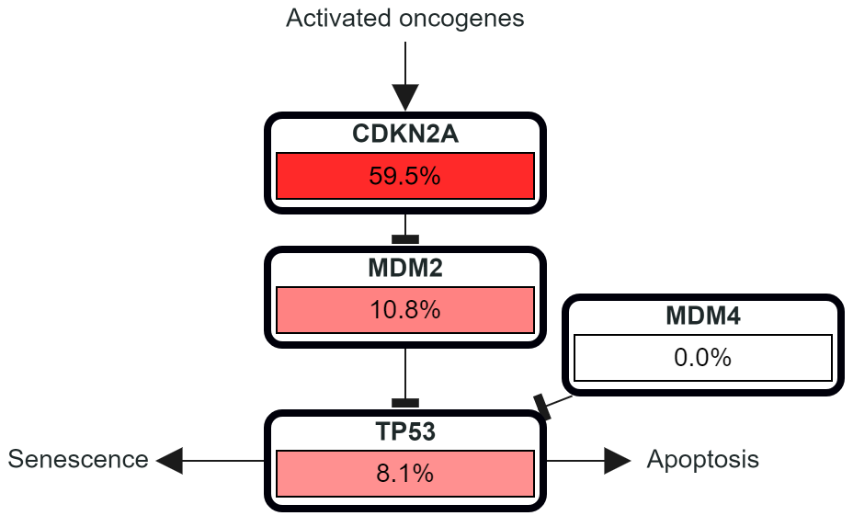

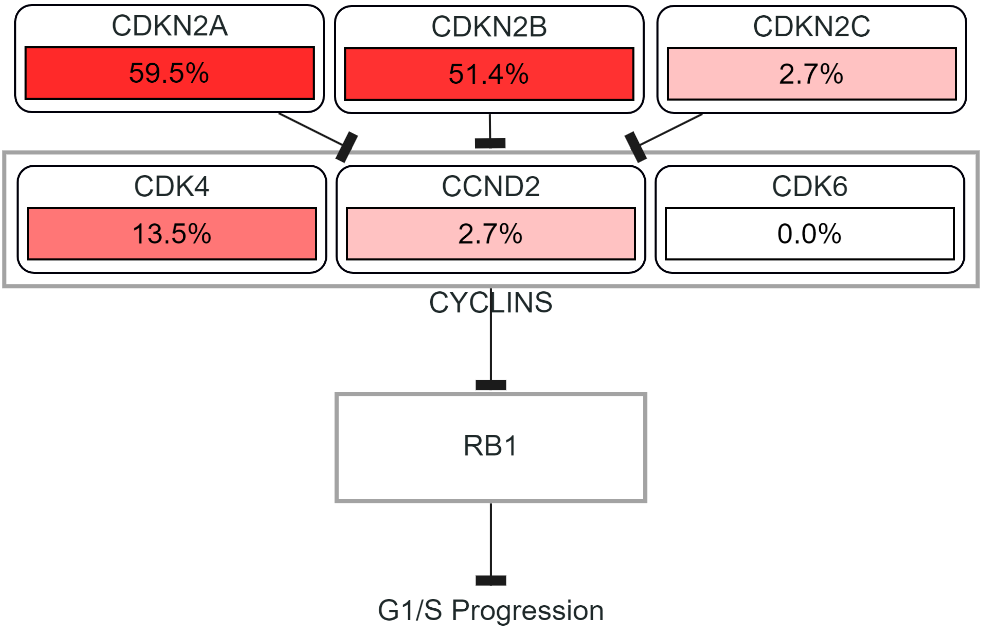


**Note:** The diagrams were created with PathwayMapper, a visual editor for summarizing cancer signalling pathway data [4].

# Supplementary Table 1. Heidelberg methylation-based classification of glioblastomas with and without *FGFR3-TACC3* fusions.

|  | ***FGFR3*-*TACC3* Fusion** | |
| --- | --- | --- |
| **Methylation Class** | **Present (n = 30)** | **Absent (n = 93)** |
| Anaplastic pilocytic astrocytoma | 0 (0%) | 2 (2.2%) |
| Diffuse midline glioma H3 K27M mutant | 0 (0%) | 2 (2.2%) |
| Glioblastoma, IDH wildtype, H3.3 G34 mutant | 0 (0%) | 1 (1.1%) |
| Glioblastoma, IDH wildtype, subclass mesenchymal | 15 (50.0%) | 39 (41.9%) |
| Glioblastoma, IDH wildtype, subclass midline | 0 (0%) | 2 (2.2%) |
| Glioblastoma, IDH wildtype, subclass RTK I | 1 (3.3%) | 19 (20.4%) |
| Glioblastoma, IDH wildtype, subclass RTK II | 14 (46.6%) | 28 (30.1%) |

**Note:** Cases were assigned DNA methylation-based classifications according to the method described by Capper et al. [10].
